# Free Sugar Consumption and Obesity in European Adolescents: The HELENA Study

**DOI:** 10.3390/nu12123747

**Published:** 2020-12-05

**Authors:** Sondos M. Flieh, Luis A. Moreno, María L. Miguel-Berges, Peter Stehle, Ascensión Marcos, Dénes Molnár, Kurt Widhalm, Laurent Béghin, Stefaan De Henauw, Anthony Kafatos, Catherine Leclercq, Marcela Gonzalez-Gross, Jean Dallongeville, Cristina Molina-Hidalgo, Esther M. González-Gil

**Affiliations:** 1 Growth, Exercise, Nutrition and Development (GENUD) Research Group, Faculty of Health Sciences, University of Zaragoza, 50009 Zaragoza, Spain; sondosnserat991@gmail.com (S.M.F.); mlmiguel@unizar.es (M.L.M.-B.); esthergg@ugr.es (E.M.G.-G.); 2 Instituto Agroalimentario de Aragón (IA2), 50013 Zaragoza, Spain; 3 Instituto de Investigación Sanitaria Aragón (IIS Aragón), 50009 Zaragoza, Spain; 4 CIBER Fisiopatología de la Obesidad y Nutrición, Instituto de Salud Carlos III, 28029 Madrid, Spain; marcela.gonzalez.gross@upm.es; 5 Department of Nutrition and Food Sciences, University of Bonn, D-53115 Bonn, Germany; pstehle@uni-bonn.de; 6 Inmunonutrition Research Group, Department of Metabolism and Nutrition, Instituto del Frío, Institute of Food Science and Technology and Nutrition (ICTAN), Spanish National Research Council (CSIC), 28040 Madrid, Spain; amarcos@ictan.csic.es; 7 Department of Pediatrics, Medical School, University of Pécs, H-7624 Pécs, Hungary; molnar.denes@pte.hu; 8 Division of Clinical Nutrition and Prevention, Department of Pediatrics, Medical University of Vienna, 1090 Vienna, Austria; kurt.widhalm@meduniwien.ac.at; 9 Inserm, U1286—INFINITE—Clinical Investigation Center—Institute for Translational Research in Inflammation and CIC 1403, University Lille, CHU Lille, F-59000 Lille, France; laurent.beghin@chru-lille.fr; 10 Department of Public Health and Primary Care, Faculty of Medicine and Health Sciences, Ghent University, 9000 Ghent, Belgium; Stefaan.DeHenauw@UGent.be; 11 Faculty of Medicine, University of Crete, GR-71003 Crete, Greece; kafatos@med.uoc.gr; 12 Food and Nutrition Research Centre—Council for Agricultural Research and Economics, 00198 Rome, Italy; catherine.leclercq@crea.gov.it; 13 ImFINE Research Group, Department of Health and Human Performance, Faculty of Physical Activity and Sport-INEF, Universidad Politécnica de Madrid, 28040 Madrid, Spain; 14 Department of Epidemiology and Public Health, Institut Pasteur de Lille, 59000 Lille, France; Jean.Dallongeville@pasteur-lille.fr; 15 EFFECTS 262, Department of Physiology, Faculty of Medicine, University of Granada, 18016 Granada, Spain; criismh@correo.ugr.es; 16 Center of Biomedical Research (CIBM), Department of Biochemistry and Molecular Biology II, Instituto de Nutrición y Tecnología de los Alimentos, University of Granada, 18071 Granada, Spain

**Keywords:** free sugars, food groups, overweight, body mass index, fat mass index, obesity, adolescents, Europe

## Abstract

Few studies have evaluated the association between dietary free sugars intake (FSI) and obesity in adolescents. We examined the relation between FSI and their contributors from the main food groups and obesity in European adolescents. We included 843 adolescents (51.6% male) from the cross-sectional HELENA study with two completed 24 h recalls and anthropometric data. Linear mixed models were applied to investigate the relation between FSI and different anthropometric indices. Odds ratios for having a high body mass index (BMI) were also estimated by multilevel ordinal regression. Total FSI was higher in males than females (102.60 g and 87.58 g, respectively, *p* < 0.001). No effect was observed between free sugar from the main food groups and BMI. Consumers of FSI from “cakes, pies and biscuits” in males (odd ratio (OR) = 0.455; 95% Confidence interval (CI) 0.251, 0.824) and from “breakfast cereals” in females had a lower probability of having obesity (OR = 0.423; 95%CI 0.204, 0.878), whereas females consuming FSI from ‘fruit and vegetables juices’ had a higher probability of obesity (OR= 2.733; 95% CI 1.286, 5.810). This study provides no evidence that increased FSI is associated with obesity in adolescents. Further studies are needed to assess the longitudinal exposure to FSI and their effect on obesity development.

## 1. Introduction

Childhood obesity is a worldwide public health concern. According to the World Health Organization (WHO), over 340 million children and adolescents aged between 5−19 years were overweight or obese in 2016 [1]. Obesity in children is linked to cardiometabolic complications that are already present during childhood [2].

Obesity could be triggered by high energy-dense food consumption [3], among other factors, in conjunction with other obesogenic risk factors, such as a sedentary lifestyle [4] and insufficient physical activity [5]. Free sugars intake has been proposed as one of the dietary contributors to obesity development in children, especially in the form of sugar-sweetened beverages (SSB) [6]. Sugar-containing soft drinks are likely consumed independent of meals and thus provide additional energy [6]. Sugars have been classified into intrinsic and extrinsic sugars [7]. Extrinsic sugars are defined as those that are not present within the cellular structure of food when consumed, which are divided into milk sugars and nonmilk extrinsic sugars (NMES). NMES refers to all mono- and disaccharides added to foods by manufacturing, cooking, and consumers, in addition to sugars that are naturally present in honey, syrup, and unsweetened fruit juice. Under these specifications, lactose is excluded, since it is naturally present in milk and milk products [8,9]. Studies have shown that diets high in NMES could result in poor diet quality, such as high energy density [10]. Noteworthily, the term “NMES” is broadly synonymous with free sugars, and these terms have been adopted in research worldwide [11]. Most countries take into account the WHO recommendations for free sugars intake [12]. In 2015, the WHO recommended that the daily consumption of free sugars should not exceed 10% of total energy intake (TEI) [13], while the Scientific Advisory Committee on Nutrition (SACN) from the UK recommended the free sugars intake to be restricted from 10% of TEI to 5% of TEI [14]. Different dietary surveys have observed that all population groups exceed this recommendation [15]. European nationwide surveys (1999–2013) showed that added sugars contributed between 11–17% of total energy intake in children [16].

Recent studies have shown that soft drinks, confectioneries, biscuits, and cakes are the main sources of free sugars in Europe in all age groups [16]. In European children, the contribution of free sugars to the total energy intake is higher than recommended, and the main food sources are sweetened beverages such as fruit juices and soft drinks [16,17]. In European adolescents from the HELENA study, free sugars intake was 110.1 g/day (19% of total energy intake; 24 h recall), while the main food contributor to free sugars intake was carbonated, soft, and isotonic drinks, followed by nonchocolate confectionaries, honey, jam, and syrup [18].

Te Morenga [19] assessed the role of free sugars on obesity development and concluded that free sugars are one of the key determinants for body weight gain in children and adults. High intake of food items containing free sugars or SSB often leads to an excess energy intake, which, if not compensated by energy expenditure, will result in an increase of body fat [19]. Several studies have found that a diet rich in SSB beverages results in weight gain or obesity [6,20,21]. However, other studies have found an inverse relation between dietary sugars intake from milk and fruits and overweight and obesity in children and adolescents, especially in females [22]. In the same vein, it was observed that free sugars from liquid sources resulted in higher body mass index (BMI), while solid foods sources alone did not have any adverse impact on obesity parameters in adults [23].

To our knowledge, there is scarce information on the association between free sugar intake and the risk of obesity development in adolescents. Thus, the aim of this study was to investigate the impact of consumption of total free sugars and their intake from the main food sources on body composition and obesity among European adolescents.

## 2. Materials and Methods

### 2.1. Study Design

The Healthy Lifestyle in Europe by Nutrition in Adolescence Cross-Sectional Study (HELENA-CSS) was a cross-sectional multicenter study (2006−2007) based on adolescents aged between 12.5–17.5 years, in 10 European cities: Heraklion and Athens (Greece), Dortmund (Germany), Ghent (Belgium), Lille (France), Pécs (Hungary), Rome (Italy), Vienna (Austria), Stockholm (Sweden), and Zaragoza (Spain) [24]. The HELENA-CSS basic objective was to obtain comparable and reliable data from selected European adolescent groups (*n* = 3528, 1845 females) using widely relevant health and nutrition-related parameters, including anthropometric measurements, physical activity, dietary intake, food choices and preferences, physical fitness, lipid and glucose metabolism, serum vitamin and mineral status, and genetic markers [25]. The inclusion criteria were participants that were not concurrently involved in another clinical trial, ages less than 17.5 years or greater than 12.5 years, and being free from any acute infection lasting less than 1 week before the inclusion process [26]. More details about the recruitment and sampling process have been described elsewhere [26].

The study was approved by Research Ethics Committees in each involved city and followed the ethical guidelines of the Declaration of Helsinki 1964: Revision of Edinburgh 2000 [27]. The ethical approval code from the coordinator center was 03/2006, obtained from the Ethical Committee of clinical research in Aragon (CEICA). Written informed consent was obtained from participating adolescents and their parents [28].

### 2.2. Study Sample

Out of the total sample of HELENA, the present study included 843 adolescents (51.6% males). The specific inclusion criteria were participants who completed two 24 h recalls with plausible intake, having complete information for weight and height, providing adequate dietary reports, and being a normal weight or overweight/obese. In this study, data of nutritional intake from eight European cities were included, because 1198 participants from Heraklion (Greece) and Pecs (Hungary) had just one 24 h recall. Adolescents who were considered over-reporters 173 (7.4%) and under-reporters 562 (24.1%) were excluded according to the approach of Goldberg et al. [29]. Additionally, 85 underweight participants were excluded from the present analysis. Also, 154 participants did not have information from accelerometers and were excluded. Finally, 513 participants did not consume food from at least one of the nine groups and, for that reason, were not included in this study.

### 2.3. Anthropometric Measurements

Body weight was measured to the nearest 0.1 kg using an electronic scale (model 871; SECA, Hamburg, Germany). Height was measured to the nearest 0.1 cm using a telescopic height-measuring instrument (model 225; SECA, Hamburg, Germany). All measurements were performed in underwear and barefoot [30]. The BMI of the participants was calculated by dividing body weight in kilograms by squared body height in meters. The obesity status was classified using the International Obesity Task Force scale [31]. Also, skinfold thickness was measured in triplicate with a Holtain Caliper (Crymmych, Wales, UK) in six body locations (right side at biceps, triceps, subscapular, suprailiac, thigh, and medial calf) and rounded to the nearest 0.2 mm. In order to predict total body fat, the Slaughter formula was used [32]. Fat mass index (FMI) was calculated by dividing body fat mass in kilograms by the square of height in meters [33]. Waist circumference (WC) was also measured three times with an anthropometric tape (SECA 200, Hamburg, Germany) at the midpoint between the lowest rib and the iliac crest [34]. The results were used as an index of abdominal fat.

### 2.4. Physical Activity Measurement

The level of physical activity (PA) was assessed using accelerometers (Actigraph MTI, model GT1M, Manufacturing Technology Inc., Fort Walton Beach, FL, USA) by placing them at the lower back, under the clothes using an elastic belt, for 7 consecutive days. The participants were given instructions to wear the instrument during their wake-up period and remove it at the time of water-based activities [35]. Using manufacturer software, data were downloaded onto a computer and analyzed later by software based on Visual Basic. The time spent (min/d) in moderate PA and vigorous PA were calculated based on cutoff points of 2000–3999 and ^3^4000 counts per minute (cpm), respectively. Time spent in moderate and vigorous PA (MVPA) was determined using the cutoff point of ^3^2000 cpm, which is equivalent to walking at 3 kph [36]. At the end of the accelerometer testing period, the participants were also asked to complete the International Physical Activity Questionnaire (IPAQ-A). This questionnaire covers four area of physical activity: (1) School-related activity, (2) transportation, (3) housework, and (4) leisure time [35]. More details have been reported elsewhere [35].

### 2.5. Questionnaires

The maternal education level of the adolescents was classified according to the International Standard Classification of Education (ISCED) and reported as primary education, low secondary education, high secondary education, and high education/university degree. In this study, the two lower levels were merged into one group called the lower level, in addition to medium and high levels [37].

### 2.6. Dietary Assessment

The HELENA Dietary Assessment Tool (HELENA-DIAT) was used to assess adolescents’ dietary consumption. This software was used as a self-administered, computerized 24 h recall, developed for Flemish adolescents and adapted and validated for use among European adolescents [38]. HELENA-DIAT is based on previous-day assessments of intake from six meal occasions (breakfast, morning snack, lunch, afternoon snack, evening meal, and evening snack). The two nonconsecutive 24 h recalls are performed on 2 convenient weekdays, 1 week apart. A well-trained dietitian was present to support the adolescents in case they need any clarification to complete the 24 h recall. To calculate energy and nutrient intakes, data from HELENA-DIAT were linked to the German Food Code and Nutrient Database (Bundeslebensmittelschlüssel, vII.3.1, Karlsruhe, Germany) [39].

The multiple source method (MSM) was used to estimate the usual dietary intake of nutrients and foods, including occasionally consumed foods [40]. The MSM first calculates individuals’ dietary intake. Then, based on their data, the MSM builds the population distribution. After applying the MSM method, dietary data were analyzed for average energy intake in kilocalories, kilojoules, and carbohydrates, including monosaccharides, disaccharides, free sugars, and total sugars in grams (g), and the percentages of their energy were calculated. The methodology described by Louie et al. [41] of the approximate added sugar content in foods was used. Free sugars content was measured by subtracting the total sugars from lactose, since the analytical data for lactose were available. Foods were grouped in order to obtain total sugars and free sugars from the most important dietary sources [18].

Nine food groups that contained free sugars were selected from the total, namely: 1—cakes, pies, biscuits; 2—breakfast cereals; 3—carbonated soft, isotonic drinks, including nonalcoholic wine and nonalcoholic beer; 4—chocolate; 5—confectionery nonchocolate; 6—milk-based desserts and puddings (including ice cream); 7—fruit and vegetable juices; 8—other sources (all other food groups); and 9—jam sugar, honey, and syrup. Since sugars in unsweetened fruit juices were categorized as natural sugars in 1980, they are, by definition, free sugars. Thus, in our results, free sugars added to fruit juices were also included [42,43].

Also, a food choices and preferences questionnaire were fulfilled by the participants, which was used to investigate the frequency of consumption of most popular snacks and drinks. The participants were asked to choose an answer based on how frequently they consumed each snack or drink, with answers ranging between never, sometimes, and often, i.e., “Do you eat sweet and candy as a snack?” (Never, sometimes, often).

### 2.7. Statistical Analysis

Analyses were carried out using IBM-SPSS (v25, SPSS Inc., Chicago, IL, USA), and Stata (v13.0, College Station, TX: StataCorp LP, USA) was used for the multilevel logistic regression, with a *p* < 0.05 representing statistical significance for all tests. Normality was assessed using the Kolmogorov–Smirnov test. Descriptive analyses mean intakes (g) and standard deviation (SD) for general characteristics and free sugars intake from various dietary sources consumed by males and females were presented. Student’s t-tests were used to compare different means by sex. ANCOVA was also used to determine the mean differences and standard deviations of free sugars from the studied food groups by BMI categories between gender, using mother’s education as covariable for all participants (consumers and non-consumers).

The residual method was used to control for the influence of free sugars on total energy intake and to investigate the associations between total free sugars itself and body composition indices. Therefore, linear regression was applied between total free sugars intake and energy intake to obtain free sugar residuals. Mixed models were used to assess the relation between total free sugars intake and body composition indices. The basic model was only adjusted for age and country, while total free sugar residual was used in the adjusted model along with age, country, mother’s education, physical activity, and total energy intake. Linear regression models were used to investigate the relation between free sugars coming from the main food groups and BMI and FMI between gender and were adjusted for age, country, mother’s education, and physical activity, total energy intake, and total free sugars residuals. Results were presented as standardized beta (β) coefficients and 95% confidence intervals. Also, multilevel ordinal logistic regression (level: City) was performed between BMI categories as dependent variable and consumption categories of free sugars from the main food group contributors in both genders. Participants were divided into consumers and non-consumers based on whether or not they consumed free sugars from the different groups. Non-consumers were considered as the reference group. The multilevel ordinal logistic regression assessed the odds ratio (OR) of having higher BMI when free sugars from the selected food groups were consumed by participants while adjusting for age, physical activity, total energy intake, mother’s education, and total free sugar residual. The results were presented as OR and 95% confidence intervals. Finally, chi square was used to investigate the relation between BMI categories and the frequency of consumption of food containing free sugars as a snack by gender.

## 3. Results

### 3.1. General Characteristics of Study Participants

Sample descriptive characteristics and mean daily intake from total free sugars are presented in Table 1 by gender. A total of 843 adolescents were included in this analysis. Males had significantly higher waist circumference (*p* = 0.025), and higher percentages of obesity (4.8%) (*p* = 0.044) than females. In the same vein, females had higher FMI compared with males (*p* < 0.001). Furthermore, the results indicated that males had a higher physical activity levels than females (*p* = 0.042). In general, maternal education level was higher for males than for females, being the significant difference between groups: *p* = 0.007. Total mean (±SD) energy intake and total mean (±SD) free sugars intake were higher in males (2813.49 kcal (857.77), 102.60 g (63.74)), respectively, than in females (2156.17 kcal (533.71), 87.58 g (49.71)), respectively, with a significant *p*-value of *p* < 0.001.

### 3.2. Free Sugars Intake from Food Sources

Supplementary Table S1 shows the mean daily intake for free sugars consumers in grams from various dietary sources consumed by both genders. These results show that males had significantly higher mean intakes from “cakes, pies and, biscuits” (*p* < 0.001), “breakfast, cereals” (*p* < 0.001), “carbonated and soft drinks” (*p* < 0.001), “chocolate” (*p* < 0.001), “confectionary nonchocolate” (*p* < 0.001), “desserts puddings” (*p* = 0.006), “fruit and vegetables juices” (*p* < 0.001), “other sources” (*p* < 0.001) and “sugar, honey, and jam” (*p* = 0.001) groups compared with females.

### 3.3. Relation between Free Sugars Intake Groups and BMI Categories between Gender

Free sugars mean intake characteristics were obtained from the different food groups and body mass index categories using mother’s education as covariable (ANCOVA) for all participants (consumers and non-consumers) (Table 2). Although the mean intake of free sugars from the different food groups in adolescents with obesity was lower than in normal-weight and overweight adolescents in males and females, there was not any significant difference among these categories.

### 3.4. Association between Total Free Sugar Intake and Anthropometric Measurements

The results of the mixed model analysis with total free sugars intake are presented in Table 3. In the basic model and fully adjusted model, total free sugars were inversely associated with the FMI in females. However, males did not show any significant result for BMI or FMI.

### 3.5. Association between Free Sugar Groups Intake and Anthropometric Measurement

Table 4 illustrates the results of the multiple linear regression model using BMI and FMI as a dependent variable and free sugars intake from the free sugar groups as independent variables in both genders. In fully adjusted models, food groups did not show any significant association with BMI, while in females, “chocolate”, “fruit vegetables juices”, and “other sources” showed an inverse association with the FMI (*p*-value < 0.05).

### 3.6. The Association between BMI Categories and Free Sugars Groups Intake Categories

Finally, a multilevel ordinal logistic regression analysis was used to assess the associations between free sugars intake from the different food groups and BMI categories between gender after adjusting for age, energy intake, physical activity, and total free sugars residual, using mother’s education as covariable and city as a level. As shown in Table 5, the normal-weight male consumers of free sugars from “cakes, pies, and biscuits” had a lower probability of having obesity compared with the non-consumers (OR = 0.455; 95% CI 0.251, 0.824). However, normal-weight female consumers of “breakfast cereals” had a lower probability of being obese (OR = 0.423; 95% CI 0.204, 0.878). On other hand, “fruit and vegetables juices” female consumers had a higher probability of having obesity compared with non-consumers (OR = 2.733; 95% CI 1.286, 5.810).

### 3.7. Free Sugars Frequency Consumption as Snack and BMI Categories between Gender

Supplementary Table S2 illustrates the relation between the frequency of the consumption of foods containing free sugars consumed as snacks, by gender, according to BMI categories. A significant result was found with males in the juices group. Males who were overweight or obese tended to consume less juices as a snack compared to normal-weight males (*p* = 0.013).

## 4. Discussion

The present study found a lack of association between total free sugars intake and free sugars from different sources and body composition indices (BMI and FMI) in European adolescents. Also, analyses were performed considering known confounders and adjusting by total energy intake and free sugars residuals.

Different foods have different content of free sugars and, for this reason, they may have a different impact on obesity development. Moreover, the food matrix may also influence free sugars. For these reasons, we analyzed total free sugars intake and free sugars from different food items.

Obviously, the results clearly show that the observed intake of total free sugars is higher than the actual recommendations of WHO and SACN [12,14] and does not generally lead to obesity in adolescents. The observed intake of total free sugars is within the range of other European studies investigating adolescents’ diet, which has ranged between 14% and 21% [42,44]. On the other hand, New Zealand’s adolescents reported higher intakes (16–26%) [45]. This high consumption of free sugars in this population group could be due to environmental factors, such as the high availability of foods with high free sugars contents; snack consumption at school; psychological factors, such as knowledge level; perception of sugary drinks and individual choice; social factors, such as their family and friends [46,47]; and the fact that preferences for sweet taste are higher in children and adolescents [48]. However, a meta-analysis of longitudinal studies concluded that sugar, confectionary, and sweets consumption decreases after adolescence [49].

In this analysis, free sugars from all food groups and total free sugars intake were higher in males than in females, as it was previously observed in the total HELENA study sample [18] and other European studies [42,50,51]. Also, it has been found that adolescent males consume more free sugars, mainly from sweetened grains, beverages, table sugars, syrups, snacks and candies, and milk products than females [52]. Females usually have lower intakes of sugars because they tend to seek a slim body image, and most of them have tried dieting at least once by cutting off sugar consumption [22].

Maternal education is important for dietary behaviors, as mothers guide children to develop and learn both food choices and eating habits. This may reflect their personal attitudes, preferences, and knowledge to understand the benefits of healthy eating [53]. Several studies have assessed the relation between maternal education and free and total sugars consumption [18,54], and have found that lower maternal education level was inversely associated with intake from “sugar, honey and jam”, “carbonated soft drinks”, “cakes and pies”, and “breakfast cereals” groups.

Several systematic reviews and meta-analyses have focused on dietary sugar and body weight in children and adolescents. In these age groups, some advice to reduce SSB and other foods containing free sugars was given as an intervention trial, and researchers did not find any association between advice and BMI or BMI z-score [55,56]. Meanwhile, cohort studies in children have confirmed the link between SSB intake and the risk of obesity but have not found a consistent relation between other measures of sugars intake and adiposity [19].

In our sample of adolescents, total free sugar was inversely associated just with the FMI in females. Some studies have reported an inverse association between free sugars intake and BMI with and without energy adjustment. Williams et al. [57] observed a significant, inverse relationship between sucrose consumption and BMI at 3–4 years of age when adjusted by total energy intake. When these children were followed-up at 7–10 years of age, the relation remained negative but not statistically significant. Another study in children noticed that free sugars intake was inversely associated with BMI but was not related to body fat [58]. Similar results were found in children with obesity who consumed less free sugars than overweight or normal-weight children [59]. Results from the Australian National Nutrition Survey also reported that intakes of total, natural, and free sugars were not associated with BMI in adolescents or children [60]. Noteworthily, it seems that the combination of both excess fat and sugars predisposes children to childhood obesity [8].

Among the possible explanations for the results from previous literature and the results of this study, individuals with obesity mainly tend to restrict their intake from high-fat and high-sugar foods when they try to reduce their weight. Meanwhile, children who are overweight or obese are less likely to restrict their dietary intake compared to adults [59]. Also, subjects with obesity tend to underreport their consumption and provide socially desirable answers, even in adolescence [61]. However, in the present analysis, under-reporters were not included. Additionally, a possible explanation for the negative result in this study was the adjustment of energy intake, since it is well known that excess energy intake plays an important role in obesity development [62]. Overall, it has been found that lean, active individuals tend to select high-energy and high-sugar diets [63]. In contrast, individuals who are overweight seem to prefer diets high in fat but tend to restrict dietary sugars [64]. In the isocaloric theory, the increase of energy from sugars is followed by lower protein intake [65]. It seems that, when individuals consume a diet high in free sugars and low in fat and energy density, they do not have excess body fat or adiposity. Meanwhile, when individuals consume a diet low in free sugars and high in both fat and energy, they tend to have higher adiposity [66]. On the other hand, studies have found that satiety is higher when consuming carbohydrates, including sugars, than consuming fat, and carbohydrates suppress eating for longer periods of time [67]. However, it was found that body fat changes occur when altering consumption mediated via altering energy intake, since the isoenergetic exchange between sugars and other carbohydrates is not associated with weight change [19].

In our sample, “chocolate”, “fruit vegetables juices”, and “other sources” showed an inverse association with FMI only in females. In Korean children and adolescents, only total sugars intake from milk and fruits was inversely associated with overweight and obesity among females [22]. However, in our study, total free sugar residuals were used as a confounding variable, and no significant associations were found between free sugar from different food sources and BMI. The possible explanation is that this method completely controlled for confounding variables by total energy intake, which reduced the risk of bias resulting from categorical adjusted variables and removing the variation caused by total energy intake in the estimation results [68].

Also, in this study, we found that overweight males with obesity tended to consume less juice as a snack compared to normal-weight males. In a cross-sectional study in children, males consumed more milk, orange juice, and carbonated soft drinks than females. However, in that study, children who consumed more milk and less carbonated soft drinks were leaner, whereas females who drank more SSB were heavier [69]. In a most recent study in China, researchers observed a weak association between BMI and SSB intake after adjusting for age, sex, total energy intake, pubertal stage, and maternal education level [70]. A meta-analysis conducted in 2013 confirmed the association between SSB intake and body fat in children, while they did not find any consistent relation between total free sugars intake and adiposity in the same age group [19]. These results can be explained by the limited number of randomized controlled trials focusing on the reduction of SSB consumption, which leads to poor cooperative reporting in children. In European children, an inverse association was noticed between total sugar intake and total energy intake with z-BMI and z-FMI using the residual method. However, this study provides no indication that increased total sugar intake positively affects BMI on an energy-equivalent basis [65].

In this study, normal-weight male consumers of free sugars from “cakes, pies, and biscuits” and female consumers of “breakfast cereals” had a lower probability of having obesity compared with non-consumers. The same result was found in another study, which showed that children who consumed the least biscuits and cakes tended to be slightly heavier than others [71]. Another study found that males who consumed SSB had a lower probability of obesity [22]. However, these results may be false due to their inability to be reproduced or due to the possibility of under-reporting. Also, as we mentioned previously, people with obesity may tend to practice selective food restriction, mainly excluding this free sugar group. On other hand, this may indicate a real prediction between this free sugar group and obesity.

Moreover, in this study, female consumers of “fruit and vegetables juices” had higher tendency toward obesity. Recent meta-analyses have reported that high intake from SSB is positively associated with weight gain and obesity development [19,72]. The main explanation of this result is that SSB may lead to weight gain due to their high added sugar content (on average, it contains about 140–150 calories and 35.0–37.5 g of sugar per 12 oz serving) and low satiety effect. Also, after intake of liquid calories, incomplete reduction in energy intake is compensated during subsequent meals [73]. In addition, studies have shown that fructose from any source promotes visceral adiposity development and ectopic fat deposition [74,75].

This study has several strengths. To our best of knowledge, this is the first study to estimate free sugars intake from various food sources and their potential association with obesity in European adolescents. The sample size was selected with a wide geographical spread from eight European cities with large cultural dietary diversity. The sample collection and several anthropometric measurements were assessed using highly standardized and validated procedures to increase accuracy. Also, we excluded under-reporters and over-reporters using a validated method, which improved the quality of the data. In addition, we used the mother’s education level as a covariable and the residual method to adjust in the analysis, which is considered an isocaloric model, to account for the direct contribution of free sugars to total energy intake. One limitation of this study was that the 24 h recalls were completed during school days. Thus, the information on Fridays, Saturdays, and holidays was not included. Another limitation of this study was its cross-sectional nature, which did not allow us to assess behavior over a period of time and did not provide information in determining the cause-and-effect relationship.

## 5. Conclusions

In conclusion, European adolescents have a high intake of free sugars, but it does not seem to be associated with BMI in both genders independently of mother’s education, physical activity, and total energy intake. These results were found even after adjusting by free sugars residuals. FSI from the consumption of “cakes, pies, and biscuits” in males and from “breakfast cereals” in females was associated with a low risk of obesity, while FSI from “fruit and vegetable juices” was associated with a high risk of obesity development in females. When assessing food groups, there were no differences by obesity degree by intake and frequency. Further studies are needed to examine the prolonged exposure to free sugars from several food sources and their effect on obesity development, followed by nutritional health promotion programs to reduce free sugars intake in European adolescents.

## Figures and Tables

**Table 1:** Characteristics of the study sample and mean daily intake of energy and free sugars. Differences of mean values by gender were considered using student’s t-test analysis.

General Characteristics	Males (*n* = 435) ^1^ M ^2^ (SD)	Females (*n* = 408) M (SD)	*p*-Value
Age	14.64 (1.2)	14.51 (1.2)	0.456
^3^ BMI (kg/m^2^)	20.67 (3.24)	20.63 (2.84)	0.265
Normal weight (*n*, %)	370 (85.1%)	351 (86.0%)	**0.044**
Overweight (*n*, %)	44 (10.1%)	46 (11.3%)	
Obesity (*n*, %)	21 (4.8%)	11 (2.7%)	
^4^ WC (cm)	72.43 (7.96)	69.10 (6.58)	**0.025**
^5^ HC (cm)	88.60 (8)	91.53 (7.62)	0.280
^6^ FMI (kg/m^2^)	6.86 (4.79)	8.81 (3.45)	**<0.001**
^7^ MVPA (min/week)	811.14 (574.87)	674.98 (510.08)	**0.042**
Maternal education (*n*, %)			
Low	140 (33.5 %)	112 (29.1 %)	**0.007**
Medium	115 (27.5 %)	140 (36.4%)	
High	163 (39.0 %)	133 (34.5 %)	
Energy intake (kcal/day)	2813.49 (857.77)	2156.17 (533.71)	**<0.001**
Total free sugars intake (g)	102.60 (63.74)	87.58 (49.71)	**<0.001**
% energy from free sugars	14.26 (6.81)	16.03 (7.24)	0.278

^1^ M: Mean, ^2^ SD: Standard deviation, ^3^ BMI: Body mass index, ^4^ WC: Waist circumference, ^5^ HC: Hip circumference, ^6^ FMI: Fat mass index, ^7^ MVPA: Moderate-to-vigorous physical activity. Boldface values indicate significance, *p*-value < 0.05.

**Table 2:** Free sugars mean intake from the main contributing food groups by body mass index categories between gender for all participants (ANCOVA).

Food Groups	Males							Females						
	Normal Weight		Overweight		Obesity			Normal Weight		Overweight		Obesity		
	^1^ M	^2^ SD	M	SD	M	SD	*p*-Value	M	SD	M	SD	M	SD	*p*-Value
Cake, pies, biscuits	15.28	5.05	15.78	5.23	15.06	4.70	0.870	13.83	4.70	14.36	5.28	12.72	4.61	0.220
Breakfast cereals	16.48	4.18	17.13	4.71	16.81	4.92	0.887	14.61	3.88	15.28	3.90	14.96	4.37	0.697
Carbonated and soft drinks *	16.77	6.04	17.48	6.63	16.14	5.36	0.711	15.46	5.32	15.65	6.13	14.77	4.97	0.544
Chocolate	16.69	5.80	18.02	4.71	17.17	5.06	0.614	14.92	5.17	15.64	5.22	14.70	5.42	0.666
Confectionary non chocolate	18.20	4.38	18.17	3.83	17.30	3.18	0.558	15.90	4.06	15.78	4.27	15.52	2.90	0.632
Desserts puddings **	17.58	2.73	17.48	3.41	17.97	2.98	0.793	14.90	2.83	15.48	3.74	14.71	3.80	0.240
Fruit vegetables juices	16.34	5.69	17.01	5.98	16.44	5.70	0.932	14.78	5.02	15.02	5.84	13.78	5.91	0.272
Other sources	15.36	6.88	16.26	7.81	14.72	6.64	0.684	14.02	6.16	14.34	7.08	12.62	6.48	0.168
Sugar, honey, and jam	16.78	4.41	16.93	4.49	15.38	3.77	0.235	15.64	3.80	15.55	4.55	14.76	3.12	0.180
Total free sugars intake	102.85	63.72	104.33	64.25	90.96	64.56	0.606	95.34	57.39	94.91	62.36	78.94	58.43	0.158

^1^ M: Mean, ^2^ SD: Standard deviation. *p*-value < 0.05. * includes isotonic drinks, nonalcoholic wine, and beer; ** includes ice cream.

**Table 3:** Mixed model analysis for total free sugars intake and their association with BMI and FMI in both genders.

Models	Males				Females			
**Basic Model 1 ^1^**	**BMI**							
	**β-Coefficient**	**95% CI**		***p*-Value**	**β-Coefficient**	**95% CI**		***p*-Value**
		**Lower**	**Upper**			**Lower**	**Upper**	
Total free sugars (g)	−0.004	−0.009	0.001	0.122	−0.003	−0.009	0.002	0.273
Total energy intake (kcal/day)	0.000	0.000	0.001	0.414	0.000	−0.001	0.000	0.676
**Adjusted model**								
Total free sugars (g) *	−0.004	−0.009	0.001	0.132	−0.004	−0.010	0.002	0.169
Total energy intake (kcal/day) **	0.000	0.000	0.001	0.244	0.000	−0.001	0.000	0.402
**Basic Model 1 ^1^**	**FMI**							
	**β-Coefficient**	**95% CI**		***p*-Value**	**β-Coefficient**	**95% CI**		***p*-Value**
		**Lower**	**Upper**			**Lower**	**Upper**	
Total free sugars (g)	−0.006	−0.013	0.001	0.087	−0.009	−0.016	−0.002	**0.012**
Total energy intake (kcal/day)	0.000	−0.001	0.000	0.282	−0.001	−0.001	0.000	0.208
**Adjusted model**								
Total free sugars (g) *	−0.006	−0.014	0.001	0.115	−0.010	−0.017	−0.003	**0.005**
Total energy intake (kcal/day) **	0.000	−0.001	0.000	0.568	−0.001	−0.001	0.000	0.069

BMI: Body mass index, FMI: Fat mass index, CI: Confidence interval. ^1^ Basic model was only adjusted for age and country. * Adjusted for age, country, mother education, moderate-to-vigorous physical activity (MVPA), total energy intake, and free sugars residual. ** Adjusted for age, country, mother education, moderate-to-vigorous physical activity (MVPA), and free sugars residual. Boldface values indicate significance, *p*-value < 0.05.

**Table 4:** Multiple linear regression models for free sugars intake from various food groups and their association with body composition indices between gender.

**Food Groups**	**BMI**							
	**Males**				**Females**			
	**Model 1 ^1^**				**Model 1**			
	**β**	**95% CI**		***p*-Value**	**β**	**95% CI**		***p*-Value**
		**Lower**	**Upper**			**Lower**	**Upper**	
Cake, pies, biscuits	−0.032	−0.092	0.028	0.290	0.006	−0.062	0.073	0.873
Breakfast cereals	−0.006	−0.079	0.066	0.866	0.033	−0.059	0.125	0.484
Carbonated and soft drinks *	−0.012	−0.062	0.038	0.633	−0.022	−0.089	0.044	0.513
Chocolate	0.000	−0.053	0.053	0.996	−0.014	−0.083	0.054	0.677
Confectionary non chocolate	−0.028	−0.100	0.044	0.445	−0.015	−0.097	0.068	0.729
Desserts puddings *	0.015	−0.093	0.124	0.778	0.017	−0.084	0.119	0.737
Fruit vegetables juices	0.001	−0.054	0.055	0.980	−0.049	−0.119	0.020	0.165
Other sources	−0.014	−0.058	0.029	0.512	−0.029	−0.084	0.027	0.314
Sugar, honey, and jam	−0.059	−0.129	0.011	0.100	0.015	−0.079	0.109	0.756
**Food Groups**	**FMI**							
	**Males**				**Females**			
	**Model 1**				**Model 1**			
	**β**	**95% CI**		***p*-Value**	**β**	**95% CI**		***p*-Value**
		**Lower**	**Upper**			**Lower**	**Upper**	
Cake, pies, biscuits	−0.059	−0.145	0.028	0.185	−0.068	−0.150	0.014	0.102
Breakfast cereals	−0.032	−0.137	0.073	0.551	−0.046	−0.157	0.066	0.420
Carbonated and soft drinks *	−0.025	−0.097	0.047	0.491	−0.078	−0.158	0.002	0.055
Chocolate	−0.019	−0.095	0.058	0.633	−0.096	−0.177	−0.014	**0.022**
Confectionary non chocolate	−0.040	−0.145	0.065	0.452	−0.077	−0.176	0.023	0.132
Desserts puddings **	0.040	−0.117	0.196	0.617	−0.032	−0.154	0.090	0.607
Fruit vegetables juices	−0.006	−0.085	0.073	0.882	−0.139	−0.223	−0.056	**0.001**
Other sources	−0.038	−0.101	0.024	0.229	−0.098	−0.164	−0.031	**0.004**
Sugar, honey, and jam	−0.94	−0.195	0.007	0.069	−0.030	−0.144	0.083	0.603

β: Regression coefficient. CI: Confidence interval, BMI: Body mass index, FMI: Fat mass index. ^1^ Model 1: Adjusted for age, country, mother’s education, moderate-to-vigorous physical activity (MVPA), total energy intake, and free sugars residuals. Boldface values indicate significance, *p*-value < 0.05. * includes isotonic drinks, nonalcoholic wine and beer; ** includes ice cream.

**Table 5:** The association between free sugars consumption from the main food group categories (non-consumer vs. consumer) and BMI categories between gender using country as a level.

Food Groups	^1^ BMI Categories								
	Males					Females			
	Free Sugars Categories	Odd Ratio	95% ^2^ CI		*p*-Value	Odd Ratio	95% CI		*p*-Value
			Lower	Upper			Lower	Upper	
Cakes, pies, and biscuits	Non-consumers	Ref	Ref	Ref	**0.009**	Ref	Ref	Ref	0.975
	Consumers	0.455	0.251	0.824		1.012	0.469	2.186	
Breakfast cereals	Non-consumers	Ref	Ref	Ref	0.513	Ref	Ref	Ref	**0.021**
	Consumers	1.227	0.665	2.265		0.423	0.204	0.878	
Carbonated and soft drinks *	Non-consumers	Ref	Ref	Ref	0.904	Ref	Ref	Ref	0.564
	Consumers	0.959	0.482	1.905		0.826	0.432	1.580	
Chocolate	Non-consumers	Ref	Ref	Ref	0.358	Ref	Ref	Ref	0.473
	Consumers	0.756	0.416	1.373		0.801	0.436	1.470	
Confectionary non chocolate	Non-consumers	Ref	Ref	Ref	0.105	Ref	Ref	Ref	0.537
	Consumers	1.686	0.897	3.171		1.220	0.649	2.294	
Desserts puddings **	Non-consumers	Ref	Ref	Ref	0.585	Ref	Ref	Ref	0.371
	Consumers	1.224	0.592	2.531		0.717	0.345	1.488	
Fruit and vegetables juices	Non-consumers	Ref	Ref	Ref	0.349	Ref	Ref	Ref	**0.009**
	Consumers	1.335	0.729	2.445		2.733	1.286	5.810	
Sugar, honey, and jam	Non-consumers	Ref	Ref	Ref	0.979	Ref	Ref	Ref	0.830
	Consumers	1.009	0.558	1.824		0.930	0.478	1.808	

^1^ BMI: Body mass index, ^2^ CI: Confidence interval. Ref: Reference group. Adjusting for age, energy intake, moderate-to-vigorous physical activity (MVPA), and total free sugar residual, using mother education as a covariable Boldface values indicate significance, *p*-value < 0.05. * includes isotonic drinks, nonalcoholic wine and beer; ** includes ice cream.

## Supplementary Materials

The following are available online at https://www.mdpi.com/2072-6643/12/12/3747/s1, Table S1 Mean daily intake in grams (and standard deviation) for free sugars consumers from various dietary sources, compared by gender using the student-t test. Table S2 Frequency of consumption of food groups containing free sugars consumed as snack, according to BMI categories and sex.
