# Supplementary material for: Free Sugar Consumption and Obesity in European Adolescents: The HELENA Study
Source: Nutrients. 2020 Dec 5;12(12):3747. doi: 10.3390/nu12123747 (PMC7762126; doi:10.3390/nu12123747)
Supplement: Supplementary file 1 [file nutrients-12-03747-s001.pdf]

Table S1. Mean daily intake in grams (and standard deviation) for free sugars consumers from various dietary sources, compared by gender using the student-t test.

Food Groups	Boys		Girls		*p*-Value
	n	Mean (SD)	*n*	Mean (SD)	
Free sugars intake from cakes, pies, biscuits (g)	252	15.40 (6.70)	248	12.59 (4.90)	**<0.001**
Free sugars intake from breakfast cereals (g)	148	16.59 (7.24)	134	12.66 (5.25)	**<0.001**
Free sugars intake from carbonated and soft drinks (g)*	320	16.88 (7.18)	260	13.80 (5.23)	**<0.001**
Free sugars intake from chocolates (g)	274	16.96 (7.26)	266	13.19 (5.04)	**<0.001**
Free sugars intake from confectionary non chocolates (g)	136	18.16 (7.59)	174	14.11 (5.12)	**<0.001**
Free sugars intake from desserts and puddings (g)**	75	17.55 (6.85)	108	13.12 (5.33)	**0.006**
Free sugars intake from fruits and vegetables juices (g)	283	16.39 (6.99)	285	13.12 (4.74)	**<0.001**
Free sugars intake from other sources (g)	435	15.49 (7.05)	408	12.56 (4.99)	**<0.001**
Free sugars intake from sugars, honey, and jams (g)	153	16.73 (7.34)	123	14.16 (5.38)	**0.001**

n: number of consumers. SD: standard deviation. Boldface values indicate significance, *p*-value < 0.05. * includes isotonic drinks, non-alcoholic wine, and beer; ** includes ice cream.

Table S2. Frequency of consumption of food groups containing free sugars consumed as snack, according to BMI categories and sex.

Food Groups	Categories	Normal Weight *n* (%)		Overweight *n* (%)		Obesity *n* (%)		*p*-Value		
		Boys	Girls	Boys	Girls	Boys	Girls	Boys	Girls	Total
Dried fruit	Never	56.70%	64.50%	53.30%	74.30%	76.50%	80.00%	0.2	0.544	0.324
	Sometimes	37.70%	32.10%	33.30%	25.70%	23.50%	20.00%			
	Often	5.60%	3.30%	13.30%	0.00%	0.00%	0.00%			
Yoghurt/ yoghurt product	Never	17.50%	13.80%	33.30%	16.70%	17.60%	10.00%	0.292	0.96	0.457
	Sometimes	43.90%	45.60%	30.00%	44.40%	41.20%	40.00%			
	Often	38.60%	40.60%	36.70%	38.90%	41.20%	0.00%			
Sweet and candy	Never	12.50%	10.20%	6.50%	13.90%	11.80%	20.00%	0.241	0.501	0.29
	Sometimes	57.90%	56.90%	77.40%	63.90%	70.60%	40.00%			
	Often	29.60%	32.90%	16.10%	22.20%	17.60%	40.00%			
Chocolate	Never	14.50%	10.20%	23.30%	13.90%	23.50%	10.00%	0.263	0.961	0.344
	Sometimes	51.30%	54.10%	56.70%	52.80%	58.80%	60.00%			
	Often	34.20%	35.60%	20.00%	33.30%	17.60%	30.00%			
Biscuit and cookies	Never	13.10%	7.00%	19.40%	11.10%	11.80%	10.00%	0.63	0.185	0.304
	Sometimes	55.90%	56.00%	51.60%	72.20%	70.60%	50.00%			
	Often	31.00%	36.90%	29.00%	16.70%	17.60%	40.00%			
Cakes/ muffins	Never	20.30%	18.90%	25.80%	8.80%	35.30%	20.00%	0.341	0.517	0.527
	Sometimes	62.70%	65.00%	51.60%	79.40%	41.20%	60.00%			
	Often	17.00%	16.20%	22.60%	11.80%	23.5	20.00%			
Bowel of cereals	Never	29.80%	26.80%	32.30%	34.30%	17.60%	40.00%	0.833	0.074	0.441
	Sometimes	37.70%	37.60%	38.70%	45.70%	41.20%	60.00%			
	Often	32.50%	35.60%	29.00%	20.00%	41.20%	0.00%			
Cereals bar	Never	49.70%	46.10%	34.50%	51.40%	58.80%	33.30%	0.444	0.908	0.924
	Sometimes	37.40%	43.80%	51.70%	40.00%	35.30%	55.60%			
	Often	12.90%	10.10%	13.80%	8.60%	5.90%	11.10%			
Milk	Never	12.70%	20.10%	13.80%	20.60%	17.60%	20.00%	0.319	0.494	0.249
	Sometimes	30.40%	28.20%	41.40%	41.20%	11.80%	40.00%			
	Often	56.90%	51.70%	44.80%	38.20%	70.60%	40.00%			
Chocolate milk	Never	15.10%	19.10%	13.80%	8.30%	17.60%	30.00%	0.711	0.306	0.377
	Sometimes	46.10%	46.90%	41.40%	61.10%	58.80%	50.00%			
	Often	38.80%	34.00%	44.80%	30.60%	23.50%	20.00%			
Carbonated soft drink	Never	9.40%	10.90%	13.30%	5.60%	17.60%	10.00%	0.149	0.693	0.629
	Sometimes	42.50%	54.10%	46.70%	61.10%	64.70%	40.00%			
	Often	48.10%	35.00%	40.00%	33.30%	17.60%	50.00%			
Juices	Never	4.30%	5.00%	6.70%	0.00%	23.50%	0.00%	**0.013**	0.579	0.173
	Sometimes	39.70%	37.30%	46.70%	33.30%	29.40%	40.00%			
	Often	56.10%	57.80%	46.70%	66.70%	47.10%	60.00%			

Boldface values indicate significance, *p*-value < 0.05.
